# Supplementary material for: The histone chaperone NASP maintains H3-H4 reservoirs in the early Drosophila embryo
Source: PLoS Genet. 2023 Mar 17;19(3):e1010682. doi: 10.1371/journal.pgen.1010682 (PMC10058107; doi:10.1371/journal.pgen.1010682)
Supplement: S1 Text — (A) Sequence alignment of CG8223 with NASP homologs in other organisms. Darkening of the color indicates greater conservation. Magenta dots represent the α-N Histone H3 binding region observed in Homo sapiens NASP. Boxed region represents the gRNA target sequence for CRISPR-based mutagenesis to generate NASP mutants. (B) Representative image of DAPI stained 0-2hr AEL embryos with the percentage of stages 1–14 embryos from two replicates. (C) TMT abundance of each channel for mass spectrometry IP TMT normalized to total peptides.(D) Localization of NASP (red) in Drosophila S2 cells. DNA is stained by DAPI (blue). Scale bar, 5 μm. Graph displays intensity profiles of NASP and DAPI through a perpendicular line. (E) Localization of NASP (green) in Drosophila S2 cells. DNA replication is marked by CldU pulsing (red) and DNA is stained by DAPI(blue). Scale bar, 5 μm. Graph displays intensity profiles of NASP, CldU, and DAPI through a perpendicular line. (F) Western blot analysis of ovary extracts from the indicated genotypes with total protein loading control. Blot is cropped for Fig 2B. Fig B. Source data for Fig 2. (A)Number of embryos laid by wild type or NASP2/Df(3R)Exel6150 mothers. Each data point is representative of a biological replicate from 70 females(n = 3). Unpaired t-test was used to determine significance (p<0.05) (B)DAFC-66D copy number relative to a non-amplified control locus from stage 12 egg chambers for the genotypes listed on the x-axis. Kruskal-Wallis ANOVA was performed to determine significance (p<0.05). (C) The number of pupae on day 10 produced from virgin females with the genotypes outlined on the x-axis crossed with wild type males. Each data point is representative of a biological replicate (n = 3). Unpaired t-test was used to determine significance (p<0.05). (D) Percentage of embryos hatched laid by wild type or NASP2/Df(3R)Exel6150 mothers. Each data point is representative of a biological replicate (n = 4) and represents the hatch rate of [file pgen.1010682.s001.pdf]

Supplemental Information

A.

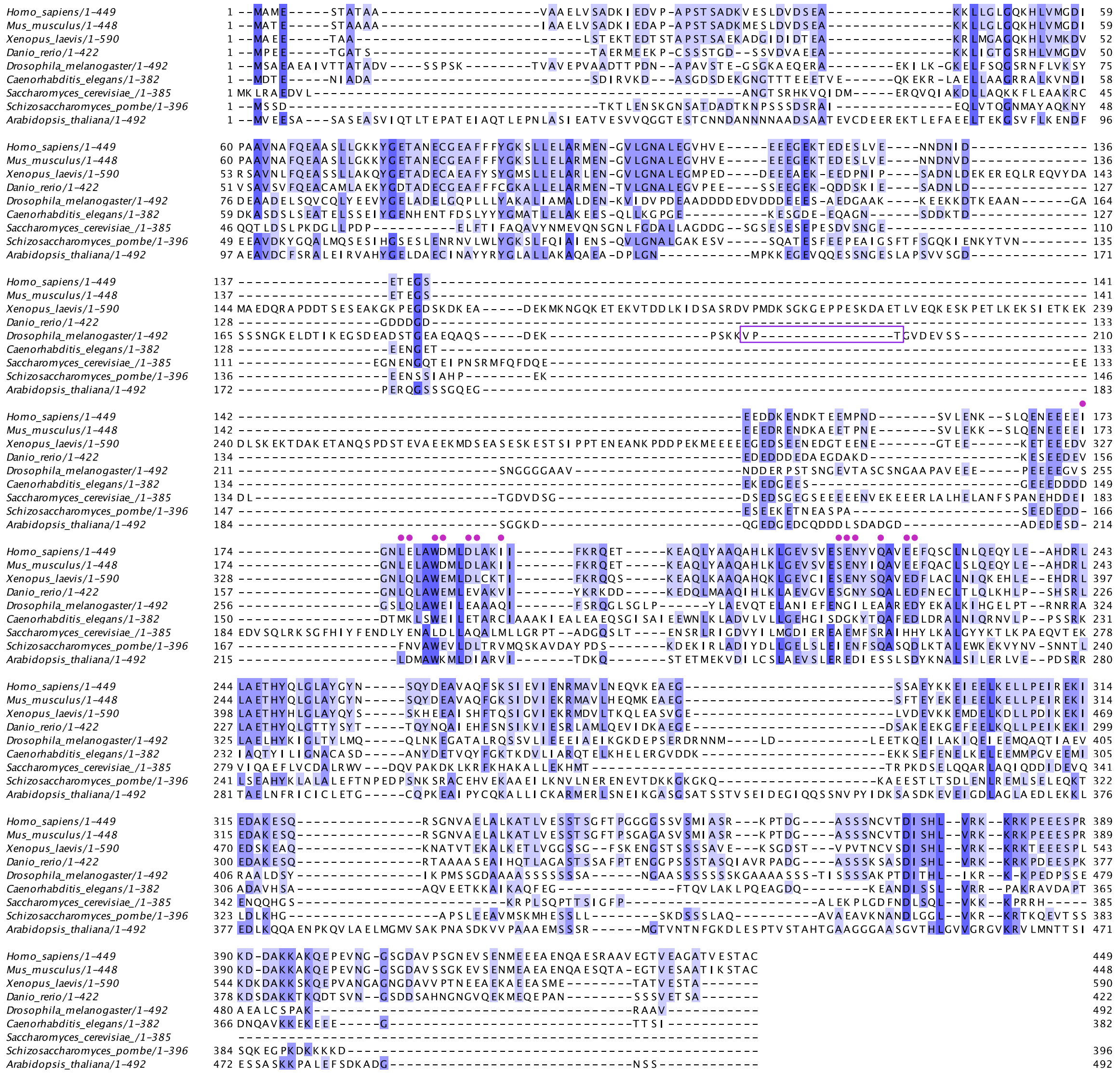

B.

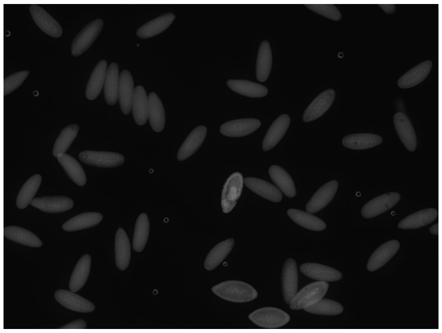

Replicate	Raw Data	%
1	47/50	94
2	48/50	96

C.

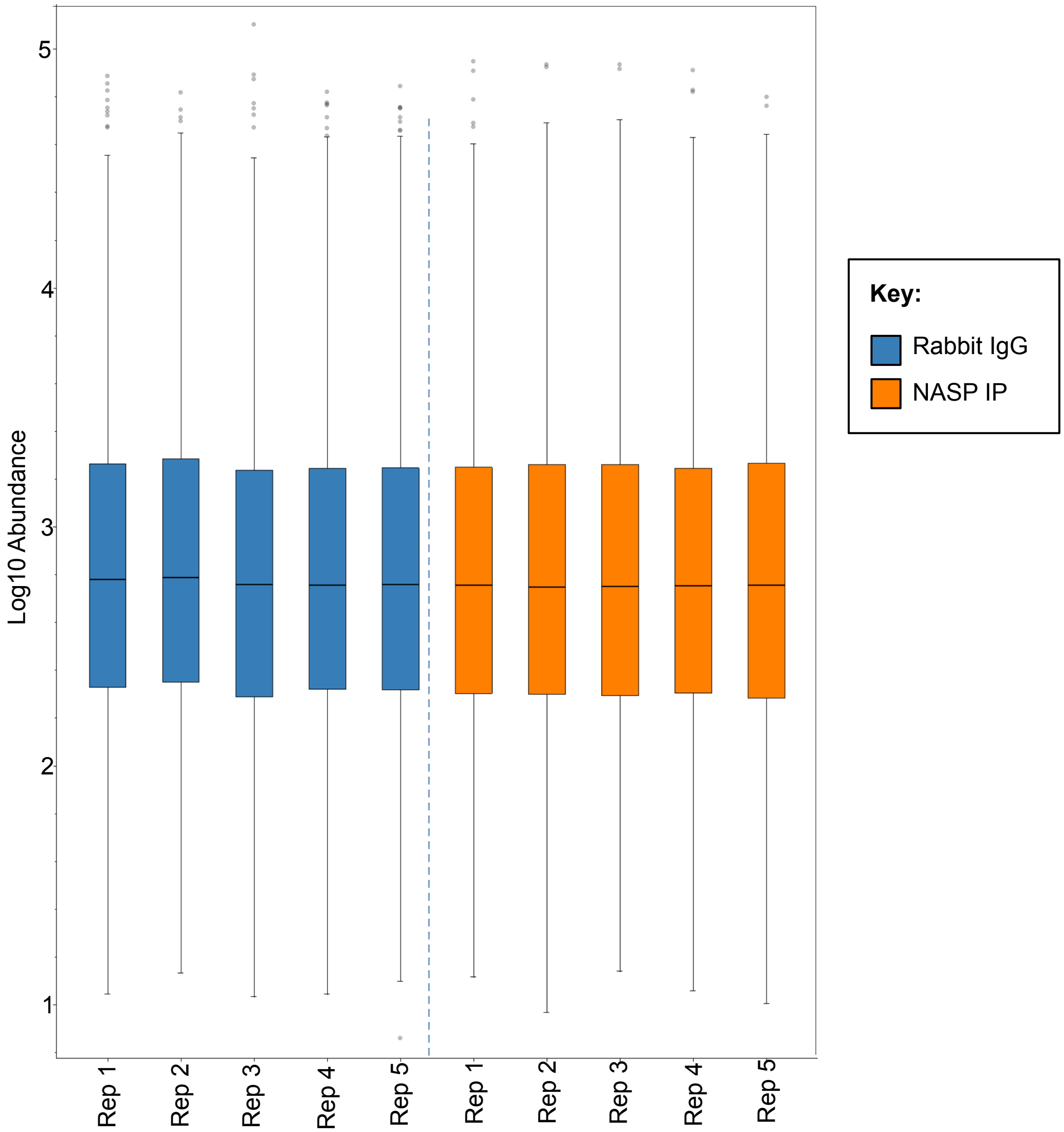

D.

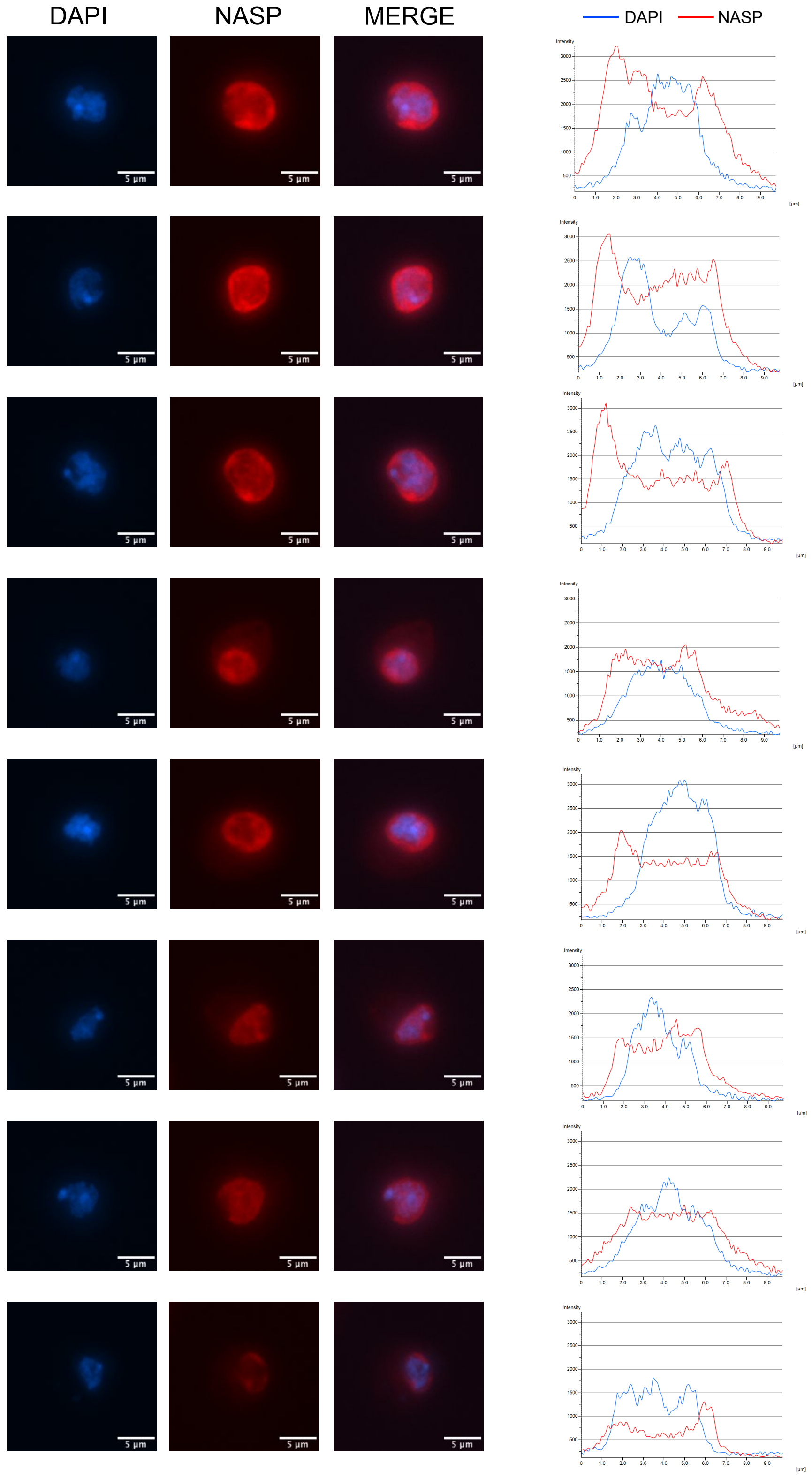

E.

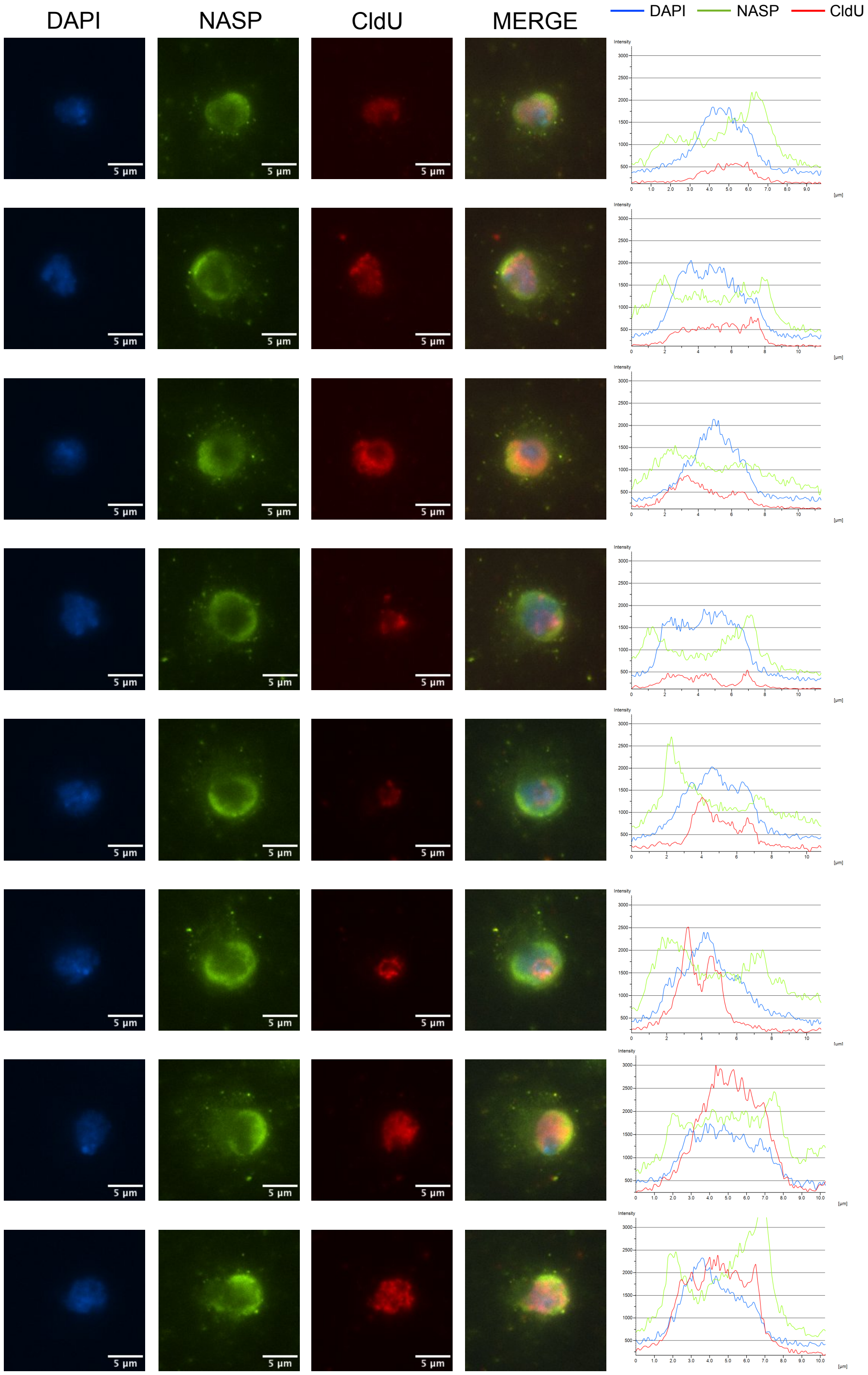

F.

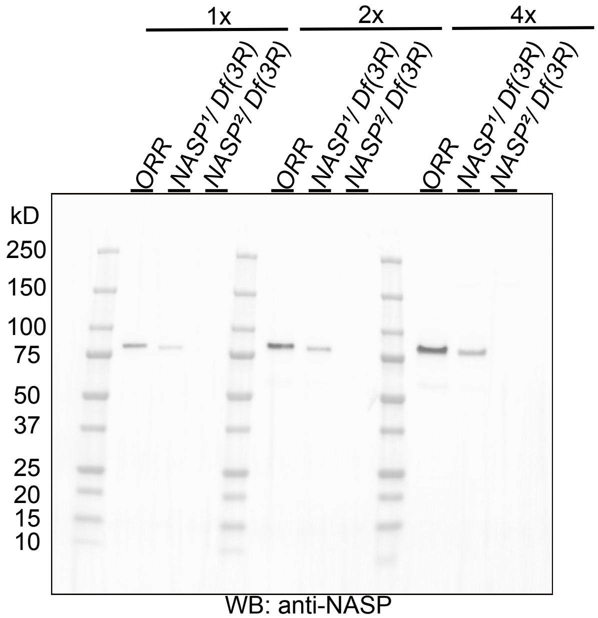

WB: anti-NASP

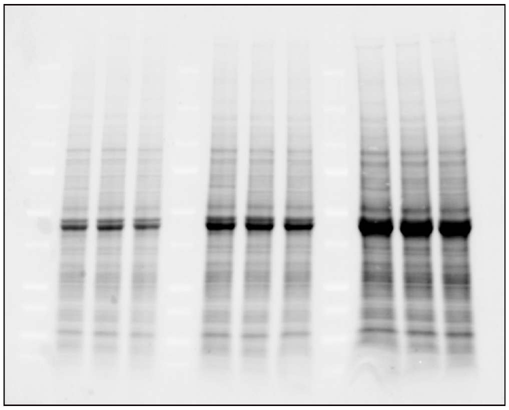

Total Protein

Fig A. Source data for Fig 1

(A) Sequence alignment of CG8223 with NASP homologs in other organisms. Darkening of the color indicates greater conservation. Magenta dots represent the α-N Histone H3 binding region observed in *Homo sapiens* NASP. Boxed region represents the gRNA target sequence for CRISPR-based mutagenesis to generate *NASP* mutants. **(B)** Representative image of DAPI stained 0-2hr AEL embryos with the percentage of stages 1-14 embryos from two replicates. **(C)** TMT abundance of each channel for mass spectrometry IP TMT normalized to total peptides.**(D)** Localization of NASP (red) in Drosophila S2 cells. DNA is stained by DAPI (blue). Scale bar, 5 μm. Graph displays intensity profiles of NASP and DAPI through a perpendicular line. **(E)** Localization of NASP (green) in Drosophila S2 cells. DNA replication is marked by CldU pulsing (red) and DNA is stained by DAPI(blue). Scale bar, 5 μm. Graph displays intensity profiles of NASP, CldU, and DAPI through a perpendicular line. **(F)** Western blot analysis of ovary extracts from the indicated genotypes with total protein loading control. Blot is cropped for Fig 2B.

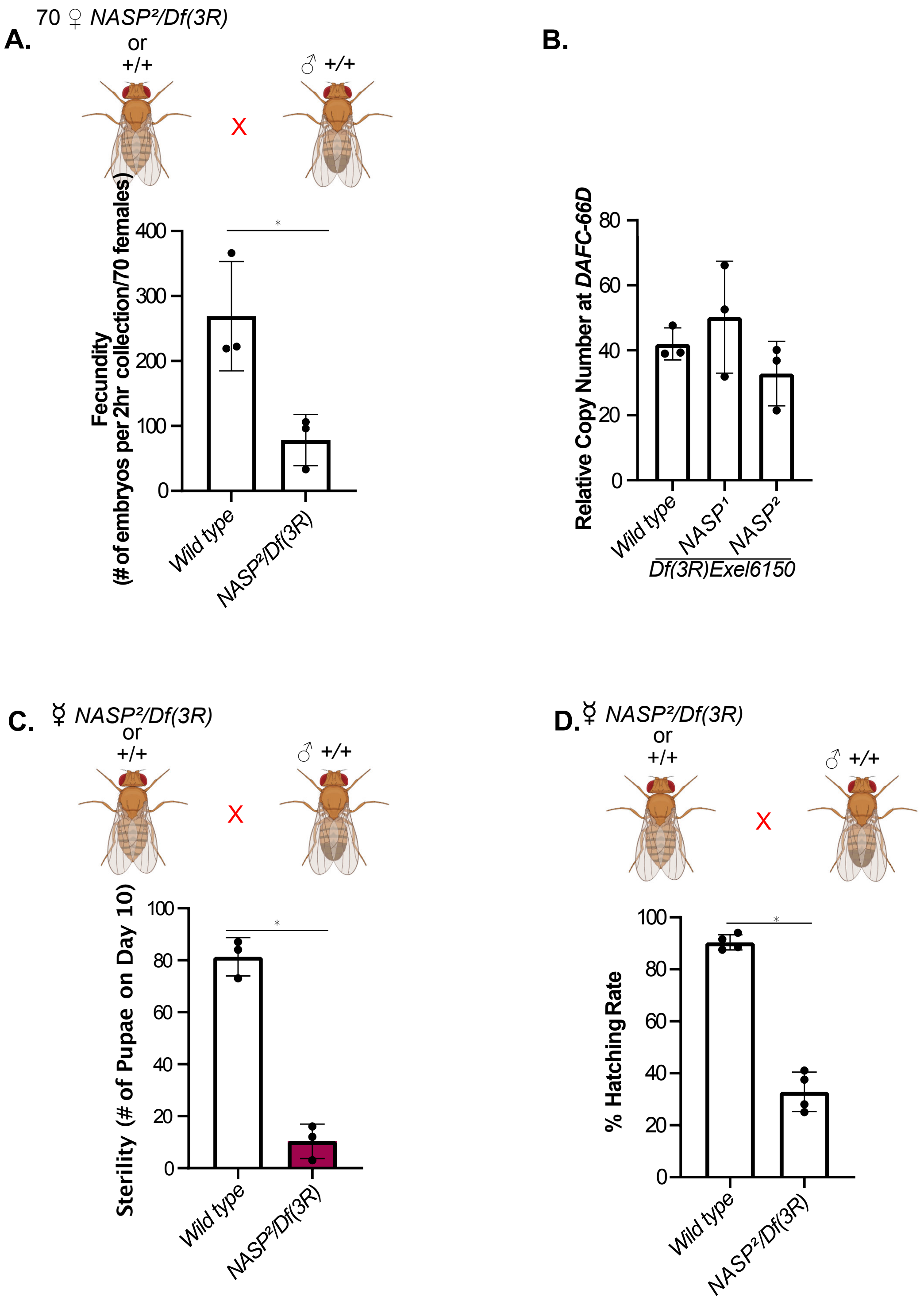

Fig B. Source data for Fig 2

(A) Number of embryos laid by wild type or *NASP*^{2}*/Df(3R)Exel6150* mothers. Each data point is representative of a biological replicate from 70 females(n=3). Unpaired t-test was used to determine significance (p<0.05) **(B)***DAFC-66D* copy number relative to a non-amplified control locus from stage 12 egg chambers for the genotypes listed on the x-axis. Kruskal-Wallis ANOVA was performed to determine significance (p<0.05). **(C)** The number of pupae on day 10 produced from virgin females with the genotypes outlined on the x-axis crossed with wild type males. Each data point is representative of a biological replicate (n=3). Unpaired t-test was used to determine significance (p<0.05). **(D)** Percentage of embryos hatched laid by wild type or *NASP*^{2}*/Df(3R)Exel6150* mothers. Each data point is representative of a biological replicate (n=4) and represents the hatch rate of a group of 100 embryos. Dunn's Multiple Comparison post-hoc was performed to determine significance (p<0.05).

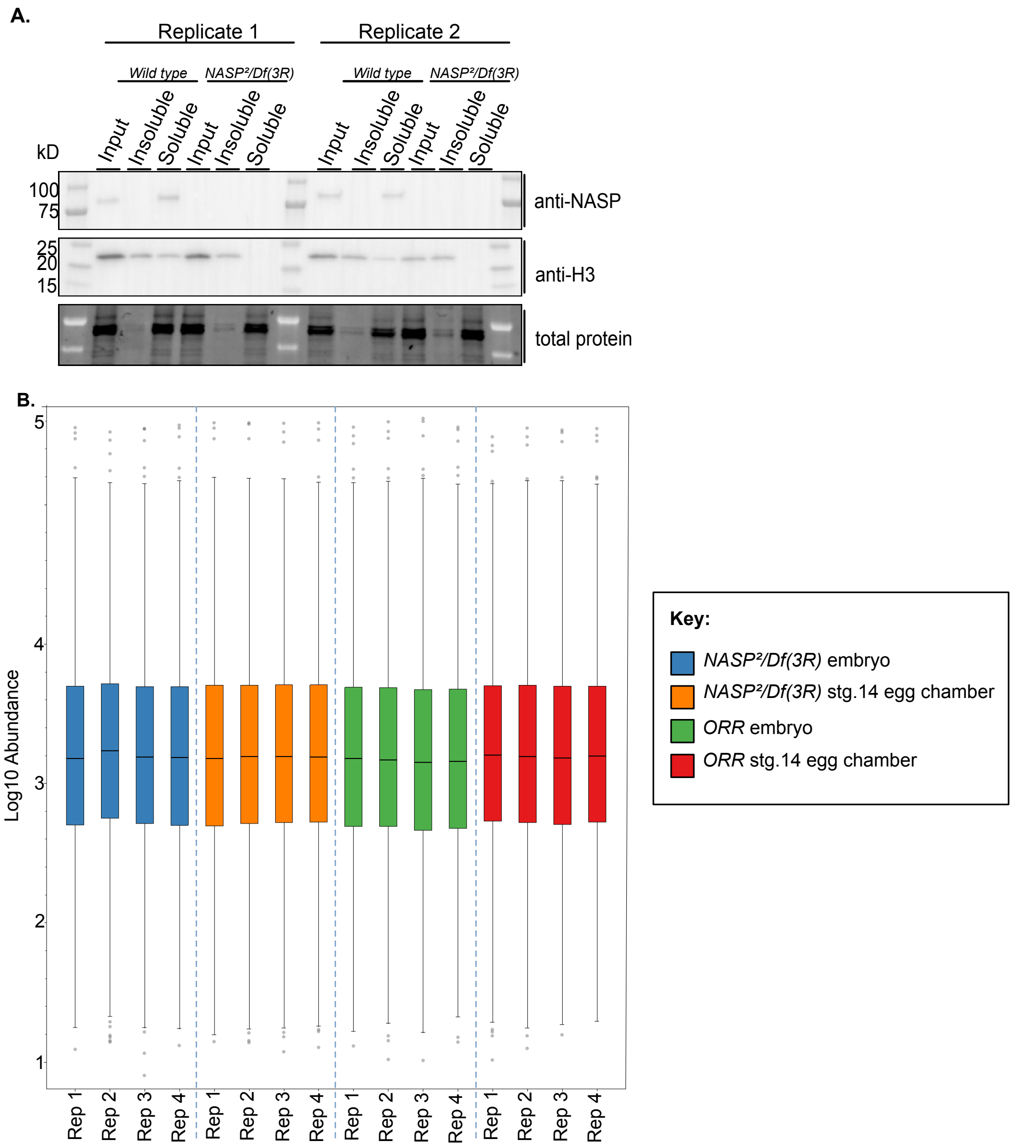

Fig C. Source data for Fig 3

(A) Western blot analysis of total, insoluble, and soluble protein preps from stage 14 egg chambers dissected from wild type or *NASP^2/Df(3R)Exel6150* mutant mothers. **(B)**TMT abundance of each channel for mass spectrometry normalized to total peptides of 0-2hr embryos and stage 14 egg chambers laid or dissected from wild type or *NASP^2/Df(3R)Exel6150* mothers.

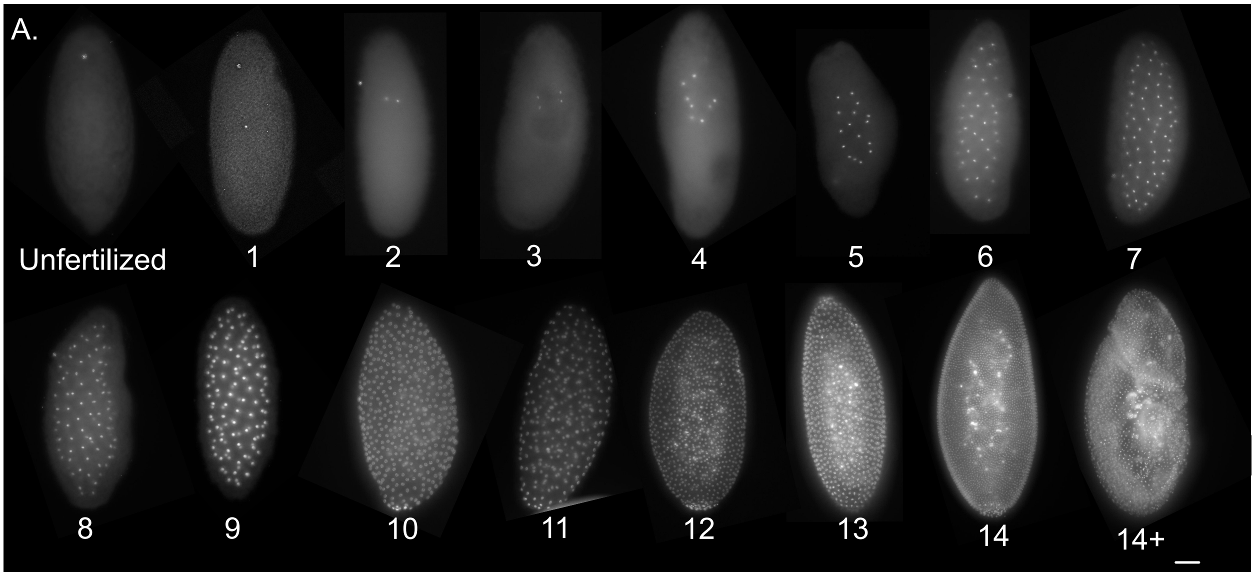

Fig D. Source data for Fig 4
(A) Representative single embryos cropped from max project images used to define nuclear cycle stages for scoring data presented in Figure 4a and 4b. Scale bar represents 100μm.
