# Supplementary material for: The histone chaperone NASP maintains H3-H4 reservoirs in the early Drosophila embryo
Source: PLoS Genet. 2023 Mar 17;19(3):e1010682. doi: 10.1371/journal.pgen.1010682 (PMC10058107; doi:10.1371/journal.pgen.1010682)
Supplement: S1 Table — (DOCX) [file pgen.1010682.s003.docx]

**Resources Table**

| Reagent | Source | Identifier | Additional information |
| --- | --- | --- | --- |
| **Antibodies** | | | |
| NASP | This Study | -- | 1:2000 |
| H3 | Abcam | ab21054 | 1:1000 |
| H2B | Abcam | ab52484 | 1:1000 |
| Peroxidase AffiniPure Donkey Anti-Mouse IgG (H+L) | Jackson ImmunoResearch | 715-035-150 | 1:20,000 |
| Peroxidase AffiniPure Donkey Anti-Rabbit IgG (H+L) | Jackson ImmunoResearch | 711-035-152 | 1:25,000 |
| Rat monoclonal [BU1/75 (ICR1)] to BrdU | Abcam | ab6326 | 1:25 |
| ALEXA FLUOR 488 GOAT Anti-Rabbit | Life Technologies | A11034 | 1:500 |
| Goat Anti-Rat IgG H&L Alexa Fluor 594 | Abcam | ab15160 | 1:350 |
| **Strains** | | | |
| Oregon R | Terry Orr-Weaver | JTN110 | -- |
| NASP^1^/TM6 | This Study | JTN403 | -- |
| NASP^2^/TM6 | This Study | JTN404 | -- |
| w[1118]; Df(3R)BSC478/TM6C, Sb[1] cu[1 | Bloomington Stock Center | 24982 | -- |
| w[1118]; Df(3R)Exel6150, P{w[+mC]=XP-U}Exel6150/TM6B, Tb[1] | Bloomington Stock Center | 7629 | -- |
| **Primers** | | | |
| CG8223 CRISPR Forward | IDT | JNpr667 | GAAGATGGAGCGGCTAAGAAG |
| CG8223 CRISPR Reverse | IDT | JNpr668 | TGGAACAAC TAGCTGTGA CCTC |
| Polymerase alpha site Forward | IDT | JNpr481 | CGCCACCTACAACAGCAGAAAA |
| Polymerase alpha site Reverse | IDT | JNpr482 | GGCTACGGTACAGGGGAGTTGA |
| DAFC-66D site Forward | IDT | JNpr581 | GCAGTGGCCTGAAAATTCTGCT |
| DAFC-66D site Reverse | IDT | JNpr582 | AGCTTAGTGCGGCAGTTTGGAA |
| **Software** | | | |
| Graphpad Prism | -- | https://www.graphpad.com/ | -- |
| Jalview | Open source | https://www.jalview.org/ | -- |
| PDB-TOOLS | Open source | https://wenmr.science.uu.nl/pdbtools/submit | -- |
| Chimera | Open source | https://www.cgl.ucsf.edu/chimera/ | -- |
| Fiji | Open source | https://fiji.sc/ |  |
