# Supplementary material for: The histone chaperone NASP maintains H3-H4 reservoirs in the early Drosophila embryo
Source: PLoS Genet. 2023 Mar 17;19(3):e1010682. doi: 10.1371/journal.pgen.1010682 (PMC10058107; doi:10.1371/journal.pgen.1010682)
Supplement: Attachment — Submitted filename: Tirgar_et_al_full-revision-plan.docx [file pgen.1010682.s005.docx]

**Manuscript number:** RC-2022-01674

**Corresponding author(s):** Jared, Nordman

*[Please use this template only if the submitted manuscript should be considered by the affiliate journal as a full revision in response to the points raised by the reviewers.*

*If you wish to submit a preliminary revision with a revision plan, please use our "*[*Revision Plan*](https://docs.google.com/document/u/0/d/1HHp62Tfeb44nd3Gu_3ChB0b8gmMdEog7GjZcZFwHSiQ/edit)*" template.* ***It is important to use the appropriate template to clearly inform the editors of your intentions.****]*

## 1. General Statements [optional]

We would like to thank the reviewers for their constructive feedback on our manuscript. By addressing the reviews, we have improved the rigor of the manuscript and provided new insight. During the revisions, we found that H3 is likely not degraded in stage 14 egg chambers, but rather forms aggregates, thus depleting the soluble pool of H3. While this analysis wasn’t requested in the reviews, we felt that it was too important not to include. This data can be found in our revised Figure 3A and 3B and Supplemental Figure 3A.

## 2. Point-by-point description of the revisions

**Reviewer #1 (Evidence, reproducibility and clarity (Required)):** 

The rapid syncytial nuclear cycles that occur during the first ~2.5 hours of Drosophila embryogenesis and give rise to the blastoderm are supported by large amounts of maternally deposited histone proteins which are stored in the egg cytoplasm for deposition into replicating DNA during each round of S phase. Although the H2A/H2B storage chaperone Jabba was identified by Michael Welte's lab several years ago, maternal H3/H4 storage chaperones have not been identified. Tirgar et al provide evidence that the Drosophila NASP protein provides histone H3 and H4 storage function during these earliest stages of Drosophila embryogenesis. The data include genetic analyses that NASP function is required maternally, but not zygotically, and molecular analyses that NASP binds H3 and that H3 and H4 levels are reduced in the embryo and late-stage oocytes in the absence of NASP. These data are convincing and support the conclusion that NASP is a maternally acting H3/H4 storage chaperone needed in the early embryo. 

Two additional lines of investigation would strengthen this conclusion and perhaps increase the impact and appeal of the manuscript.

The first is a microscopic analysis of the nuclear division cycles in eggs derived from NASP mutant mothers. The authors report DAPI staining and assessment of nuclear cycles, but do not show these data. In fact, the two embryos shown in Figure 4B do not look like DAPI stained embryos-there are no nuclei apparent in the images. Loss of maternal histone causes defects in chromosome morphology that result in characteristic defects such as lagging chromosomes and the failure of sister chromatid segregation leading to fused daughter nuclei (see PMID: 11157774 for an example). These defects should not be difficult to detect via DNA staining or even using fluorescently labeled H2 type histones. Characterizing such defects would lend support to the hypothesis and I think is important for this paper.

We thank the reviewer for their constructive review and feedback. We have switched to Propidium Iodide (PI) staining to increase the signal-to-noise for DNA staining in early embryos. Given the improved signal we see with PI over DAPI, we now have provided both improved images of nuclear staining and measured defects in chromosome morphology as suggested. This data has been included in Supplemental Figure 4A,Figure 4C, and 4D.

Second, determining the location of NASP in the early embryo might provide further insight into the mechanism of storage. i.e. is NASP located in the cytoplasm rather than the nucleus, perhaps in association with lipid droplets like Jabba? Do the antibodies the authors developed work in IF experiments to ask this question? At the moment what is shown is that NASP is present in 0-2 hour embryos via western blot analysis, supporting the conclusion that it functions in the early embryo as a storage chaperone. This analysis would be nice to have but is not essential in my view. 

We have tried to use our antibody to monitor the localization of NASP in the early embryo. We have used a wide array of fixation and permeabilization conditions but still have been unsuccessful. We have, however, been able to monitor NASP localization in Drosophila S2 cultured cells with our antibody. We have included the NASP localization data in S2 cells in combination with CldU labeling to mark cells in S phase. This new data can be found in Supplemental Figure 1E and 1F.

Small points: 
Is NASP really a maternal effect "lethal"? Some of the eggs do hatch, and so some develop to stages where maternal histones are no longer necessary and zygotic production takes over (i.e. cycle 15). Perhaps consider the language used here.

We see the reviewers point with respect to the term ‘lethal’. We do see a very small fraction of progeny laid by *NASP* mutant mothers make it to adulthood, although they die shortly after hatching. We’ve removed the term ‘lethal’ and refer to NASP solely as a maternal effect gene.

On this point, do NASP mutant females lay the same number of eggs as wild type? i.e. is there a requirement for oogenesis/egg production (other than depositing H3/H4 into the egg), or just for the early zygotic cycles? 

We have noticed that *NASP* mutant mothers have lower fecundity. We have included this data in the revised manuscript as Supplemental Figure 2A.

The first paragraph of the results is redundant with much of the introduction, which I think could do a better job at describing in more detail the syncytial cycles and the special needs they have for histone storage and chaperone function versus the post-blastoderm embryonic cycles and the rest of development. i.e. make a better distinction between the first two hours of embryogenesis versus the rest of embryogenesis, and the when the switch from maternal to zygotic control of development and histone production occurs (cycle 15 at 3-4 hours AED).

We appreciate the reviewer for this suggestion. The manuscript has been edited to be less redundant and include details of embryogenesis as suggested.

CROSS-CONSULTATION COMMENTS 
Seems like all reviewers are in general agreement, particularly about providing additional data regarding chromosome/nuclear behavior in the NASP mutants and NASP localization in the early embryo to increase impact of the study. While rescue of the NASP mutant phenotype with a transgene would be nice, as suggested by referee #2, I don't think it's essential given the genetic approaches employed. 

Reviewer #1 (Significance (Required)): 

see above 


**Reviewer #2 (Evidence, reproducibility and clarity (Required)):** 

Tirgar et al. report on a functional characterization of the Drosophila homolog of the histone H3/H4 chaperone NASP. They generated a loss of function allele of NASP by CRISPR/Cas9, which induces a partial maternal effect embryo lethal phenotype. Using quantitative mass spectrometry, they demonstrate that NASP stabilizes reservoirs of H3 and H4 in the early embryo. 
The manuscript is very clear and confirms the functional importance of maternal NASP for the early embryo. Genetic analyses are well conducted (but see my comments below) and the impact of NASP maternal mutant on H3 and H4 stockpiles is convincingly established by both quantitative mass spectrometry and Western-blotting. 

Major comments: 

- Although the authors used two independent deficiencies of the NASP genetic region to characterize their NASP CRISPR alleles, it is relatively standard in this type of functional analyses to perform rescue experiments using a transgene expressing the WT protein.

We thank the reviewer for this suggestion. As discussed in the cross consultation, we agree that the use of the two different deficiency lines and the *NASP^1^* CRISPR control are clear lines of evidence that the phenotypical data are due to lack of *NASP*.

- In WB analyses, NASP appears systematically shorter in the NASP[1]/Df genotype compared to WT. Can the authors comment on this?

While we reproducibly see this change in migration, we can only guess as to why this may be. One possible reason is that the NASP^1^ mutant protein could be missing a post-translational modification. Proteomic data from Krauchunas *et al. (Dev Biol.* 2012; PMC3441184) shows that NASP has the potential to be regulated by phosphorylation. Therefore, the NASP^1^ mutant protein could be missing a phosphorylation. Intriguingly, the 6bp insertion is next to a Thr residue that could affect its ability to be phosphorylated (if it is phosphorylated at all). Since we can only offer speculation, we do not feel comfortable adding this to the manuscript.

- The authors do not mention the centromeric histone H3 variant Cid in their analyses. Do they have evidence that it is not affected by loss of maternal NASP?

We thank the reviewer for raising this great point. Our mass spec data reveals that Cid levels stay the same in the absence of NASP in both embryos and stage 14 egg chambers. We have edited Figures 3D and 3E to include Cid. Unfortunately, we did not identify any Cid-specific peptides in our IP-mass spec data.

- The authors could have chosen to explore in more details the phenotypic defects of embryos derived from NASP mutant mothers. Instead, a single abnormal embryo is shown with no cytological details. This is a bit problematic since an earlier study (Zhang et al 2018, cited in the manuscript) actually provided more phenotypic details of embryos from NASP KD mothers.

This issue was also raised by Reviewer 1. We have switched to Propidium Iodide (PI) staining to increase the signal-to-noise for DNA staining in early embryos. Given the improved signal we see with PI over DAPI, we are now able to provide both improved images of nuclear staining and assay for defects in chromosome morphology as suggested. This data has been included in Figures 4C and 4D. We also have shown representative embryos so readers can see how we were able to stage embryos (see Supplemental Figure 4A).

We would like to note that the data provided by Zhang et al, 2018 used a single RNAi line to target CG8223. There were no controls for off-target effects nor was a second RNAi line used to confirm the phenotype was specific to CG82232 knock down.

- Similarly, the authors could have used their anti-NASP antibody to analyze the distribution of NASP during cleavage divisions. Does it behave like ASF1, for instance, which enters S phase nuclei at each cycle or does it remain in the cytoplasm? These are relatively simple experiments/analyses that could increase the significance of the study. 

This point was also raised by Reviewer 1. We have tried to use our antibody to monitor the localization of NASP in the early embryo. We have used a wide array of fixation and permeabilization conditions but still have been unsuccessful. We have, however, been able to monitor NASP localization in Drosophila S2 cultured cells with our antibody. We have included the NASP localization data in S2 cells in combination with CldU labeling to mark cells in S phase. This new data can be found in Supplemental Figures 1E and 1F.

Minor comments: 

- line 60: I suggest to introduce Drosophila in the next sentence, where it seems more appropriate (not all embryos develop "extremely rapidly").

We have edited the second sentence to state “the early Drosophila embryo”.

- line 68: the 50% estimation of free histones does not really make sense without defining the embryonic stage.

We have edited the manuscript to state the specific cell cycle in which there has been 50% free histones measured.

- line 89: Are the authors specifically referring to Drosophila NASP?

Yes, we have edited the text to include Drosophila in this instance.

- lines 99-106: I found this paragraph redundant with the introduction.

We appreciate this suggestion. It was also pointed out by Reviewer 1. We have made changes to the manuscript to address the redundancy.

- line 142: H3-H4

Thank you for noticing this. We have edited the text to include 4.

- line190-191: It seems to me that data of Figure S2C are already included in Fig. 2E.

The data in Figure S2C was performed with virgin females compared to the data in Figure 2E that was generated with non-virgin mothers. This was important to control the genotype of the embryos.

- line 232: it is surprising that the Zhang et al paper (reporting maternal KD of NASP) is only mentioned here. As a reader, I would certainly prefer to have it presented right from the introduction.

We have edited the manuscript to include this reference in the introduction.

- Figure 4B needs a scale bar.

Figure 4B has been replaced. We have now included scale bars on all images.

- line 302: Mentioning the identity and function of known H3/H4 histone chaperones acting in the early embryo (ASF1, HIRA, CAF-1, ...) could provide perspective to the present study.

Thank you for this suggestion. We have edited the manuscript to include functions of other histone chaperones in the early embryo to provide context.

- line 304: in contrast to this statement, I found quite surprising and interesting that NASP is not absolutely essential for embryo development considering its role. This should be discussed.

In the absence of Jabba alone, upregulation of translation can compensate for the destabilization of H2A, H2B, and H2Av. It is only when translation is inhibited in embryos laid by *Jabba* mutant mothers that embryos die (Li.Z, *et al. Curr Biol* 2013). Therefore, it is possible that translation can partially compensate for the degradation of H3 and H4 in the absence of NASP. This may be why a fraction of embryos laid by NASP mutant mothers are able to hatch and why we still detect some H3 in embryos laid by *NASP* mutant mothers. We have edited the manuscript to discuss this more in depth.

CROSS-CONSULTATION COMMENTS 
I fully agree with the other reports. 
The NASP rescue experiment is just a suggestion but is not essential. 

Reviewer #2 (Significance (Required)): 

This work clarifies the identity and function of Drosophila NASP and clearly demonstrates that NASP is important for the stabilization of maternal stockpiles of H3 and H4 during early embryo development. The conservation of NASP function as a histone H3/H4 chaperone in Drosophila is not really a surprise but the merit of this study is to establish this assumption as a fact. It also establishes useful tools (mutant lines and antibody) for the fly community interested in this topic. The study however does not provide new insights about the dynamic distribution of NASP and the cytological consequences of its maternal depletion on the amplification of cleavage nuclei. 


**Reviewer #3 (Evidence, reproducibility and clarity (Required)):** 

Summary: 
Rapid cell cycles in early embryogenesis is driven from maternally supplied stockpiles of RNA and protein, including histones H3 and H4. This study uses sequence homology searches, biochemical approaches (immunoprecipitation and mass spectrometry) and genetics to identify NASP (CG8223) as the H3-H4 chaperone in Drosophila. Using CRISPR technology, the authors generate a NASP mutant fly line and show using genetic crosses that NASP is a maternal lethal gene. Furthermore the study shows that NASP stabilises H3-H4 during oogenesis and embryogenesis and is required for early embryogenesis. 

Major comments: 
The key conclusions of this study are very convincing. For example, the authors use multiple approaches to show H3-H4 specific interactions with NASP and that H3-H4 protein levels are reduced in mutants (Western analyses, quantitative MS). Analysis is carried out on two individual NASP mutant lines (one deletion that produces no protein, one insertion that still produces some protein acting as a control). All experiments are well controlled, executed and presented. Genetic crossing schemes are well presented and statistical analysis of progeny is clear. 

We thank the reviewer for their positive feedback of our manuscript.

Minor comments: 
In Figure 1B - Authors could indicate amino acids shown or are they full length proteins?

We have edited the methods to include specific amino residues that are included for each structure.

In Figure 2B - Authors could (semi) quantify reduction in NASP1 mutant to show this is a gene dose effect?

We have now included the quantification of the Western blot in Figure 2B. We also added new Western blots in Figures 3A and 3B and included quantification of those blots as well.

CROSS-CONSULTATION COMMENTS 
I agree with the other reports. Although I did not indicate it in my original report, I agree that more in depth analysis of nuclear or chromosomal defects in NASP mutant embryos would enhance the study.

Thank you for this suggestion. We have repeated all the DNA staining with propidium iodide and have shown representative images of embryos so readers are confident with our staining and staging (see Supplementary Figure 4A).

Reviewer #3 (Significance (Required)): 

Excess soluble histones can be toxic and must be bound to chaperones. Until this study the chaperone responsible for H3-H4 stabilisation in rapidly cycling cells in Drosophila embryos was not known. Moreover, the NASP homolog had not yet been identified in Drosophila nor had its function been characterised. The findings are of interest to Drosophila researchers, the field of chromatin assembly, as well as those interested in early embryogenesis in animals.

## 1. Description of the revisions that have already been incorporated in the transferred manuscript

*Please insert a point-by-point reply describing the revisions that were already carried out and included in the transferred manuscript. If no revisions have been carried out yet, please leave this section empty.*

- All of the suggested textual edits have been made and are highlighted in the revised manuscript.
- We have added Supplemental Figure 2A showing the lower fecundity of *NASP* mutant mothers.
- We have included Cid data in Figures 3D and 3E.
- We have edited Figure 2B to include a quantification of NASP band intensity.
- We have expanded the Western blot analysis of histone levels in Figure 3A and 3B.
- We have completely repeated the cell cycle analysis in Figure 4

## 2. Description of analyses that authors prefer not to carry out

*Please include a point-by-point response explaining why some of the requested data or additional analyses might not be necessary or cannot be provided within the scope of a revision. This can be due to time or resource limitations or in case of disagreement about the necessity of such additional data given the scope of the study. Please leave empty if not applicable.*

After cross consultation, the reviewers have agreed that the rescue experiment with a *NASP* transgene was not essential. We are confident that the phenotypes described are due to the absence of *NASP* because we used two different deficiency lines and the *NASP^1^* CRISPR control. Further, it would take ~4 months to generate the fly line and perform this experiment.
