# Supplementary material for: The histone chaperone NASP maintains H3-H4 reservoirs in the early Drosophila embryo
Source: PLoS Genet. 2023 Mar 17;19(3):e1010682. doi: 10.1371/journal.pgen.1010682 (PMC10058107; doi:10.1371/journal.pgen.1010682)
Supplement: Attachment — Submitted filename: Reviewer Response.docx [file pgen.1010682.s006.docx]

Reviewer #1: no

Reviewer #2: Summary:
The rapid early embryonic cycles in Drosophila pose a need for maternal histone supply, as well as histone stabilisation by specific chaperones before deposition. This manuscript investigates the H3-H4 chaperone NASP homolog in Drosophila. Based on multiple lines of evidence - sequence homology, structural predictions, and biochemical co-purification with H3-H4 (by IP/MS) - the authors convincingly identify gene CG8223 as Drosophila NASP. Functional experiments show that soluble H3-H4 pools are destabilised in the absence of CG8223 and provide further support. NASP deletion by CRISPR effectively demonstrates that the null mutant is viable (using various deficiency lines). Moreover, NASP is a maternal effect gene and NASP mutant mothers have defects in embryonic development and embryo hatching. This study identifies the fly NASP homolog and effectively demonstrates its functional requirement in embryo development.

This revised version of the manuscript addresses most, if not all, of the previous concerns raised. I have three additional comments/suggestions:

1. The more in-depth characterisation of nuclear and segregation defects in early embryos now provided in Figure 4 strengthens the study. Also, the additional discussion of previous findings from Zhang et al 2018 provides clarity on the novelty of this current study. Related to this phenotype, the authors suggest the possibility in the Discussion that embryos laid by NASP mutant mother are not fertilised or develop as haploids. Is it not possible to already decipher this information from the images collected for quantitation in Figure 4?

We thank the reviewer for their comments. While we appreciate this suggestion, due to variability in PI staining intensities between embryos, we do not feel comfortable measuring ploidy by PI intensities between different samples. It is a question we will pursue by other methods in the future.

2. With respect to NASP localisation, the authors were unable to confirm its localisation in embryos. They do however present results showing its localisation in cultured S2 cells, where it appears to be enriched in the cytoplasm. Some further discussion of this localisation pattern and its predicted localisation in the syncytium would be useful to explain/predict its function in embryos. Also, some further explanation of the observed localisation in S phase would be useful.

We like to emphasize that the localization in Drosophila cultured S2 cells is at the periphery of the nucleus and not necessarily in the cytoplasm. Further, in our experiments, we do not observe any obvious difference in NASP localization during S phase. Given that we do not have any data addressing the pattern of NASP localization in syncytial embryos, we feel it is premature to speculate.

3. Based on Figure 3, the authors claim that H3 forms insoluble aggregates in stage 14 egg chambers and propose a specific role in solubilisation at this stage. IF images of H3-H4 localisation in stage 14 egg would significantly strengthen this claim.

We appreciate the reviewer for this great idea. Unfortunately, we were unable to monitor H3 (or H4) localization in any stage egg chamber. Based on this suggestion, we tried the experiment using the antibodies available in our lab. Unfortunately, IF using either the H3 or H4 antibodies were not successful. We are, however, generating fly lines with tagged H3 for live imaging. We hope this approach will be more fruitful.

Also, there appears to be an error in the labelling of lanes in Fig S3A.

We thank the reviewer for noticing this error. The last lane of replicate two of this experiment was accidentally double loaded with the MW ladder. We have uploaded a revised blot with two new replicates that are now properly labelled (and loaded).

Minor:
Line 55: remove ‘provides’

Thank you for noticing this. We have removed ‘provides’.

Reviewer #3: The authors have addressed all my suggestions and I only have a few formal comments below (one is quite important, though). Congratulations on this fine work!

- These two sentences of the abstract are a bit redundant and could possibly be merged into one : « Here, we show that CG8223, the Drosophila homolog of NASP, is a H3-H4-specific chaperone in the early embryo. NASP specifically binds to H3-H4 in the early embryo. »

Thank you for this suggestion. We have removed the second sentence.

- In the sentence « Interestingly, we noticed a higher level of H3
and H3.3 in CG8223 IPs relative to H4, suggesting that CG8223 preferentially binds to H3 », I understand that H3 first designates H3.2 (the replicative Drosophila H3) and then is used as a generic name for H3. To avoid confusion, the authors could use H3.2 when it is relevant.

We thank the reviewer for this suggestion. We have included a clarification sentence to let the readers know that H3 relates to the replicative Drosophila H3.2.

- Important point : in the discussion, the authors speculate on the possibility that defects observed in developing NASP embryos could result from a fertilization failure. This is actually not possible because fertilization is essential to initiate embryogenesis in Drosophila (the sperm provides centrioles to the egg). In embryos from Hira mutant females, eggs are fertilized but the male pronucleus is not properly formed. The corresponding paragraph should thus be rephrased.

We thank the reviewer for making this important distinction. We have edited the discussion to clarify that a subset of the embryos laid by NASP mutant mothers are either unfertilized OR are fertilized and could develop as haploids, similar to the HIRA mutant.

**Changes:**

Supplemental Figure 3A has been replaced with two new replicates.

The color bar on the 12-14+ legend for Figure 4B has been corrected to match the color of the graph.

All text edits are tracked on manuscript.
